# Regulatory considerations for developing a phase I investigational new drug application for autologous induced pluripotent stem cells‐based therapy product

**DOI:** 10.1002/sctm.20-0242

**Published:** 2020-09-18

**Authors:** Balendu Shekhar Jha, Mitra Farnoodian, Kapil Bharti

**Affiliations:** ^1^ Center for Cell Engineering, Department of Transfusion Medicine Clinical Center, National Institutes of Health Bethesda Maryland USA; ^2^ Ocular and Stem Cell Translational Research Section, Ophthalmic Genetics and Visual Function Branch National Eye Institute, National Institutes of Health Bethesda Maryland USA

**Keywords:** GLP, GMP, IND, phase I clinical trial, preclinical work

## Abstract

Induced pluripotent stem cells (iPSC)‐based therapies have been hailed as the future of regenerative medicine because of their potential to provide treatment options for most degenerative diseases. A key promise of iPSC‐based therapies is the possibility of an autologous transplant that may engraft better in the longer‐term due to its compatibility with the patient's immune system. Despite over a decade of research, clinical translation of autologous iPSC‐based therapies has been slow—partly due to a lacking pre‐defined regulatory path. Here, we outline regulatory considerations for developing an autologous iPSC‐based product and challenges associated with the clinical manufacturing of autologous iPSCs and their derivatives. These challenges include donor tissue source, reprogramming methods, heterogeneity of differentiated cells, controls for the manufacturing process, and preclinical considerations. A robust manufacturing process with appropriate quality controls and well‐informed, prospectively designed preclinical studies provide a path toward successful approval of autologous iPSC‐based therapies.

## Graphical abstract

Regulatory considerations for developing a phase I investigational new drug application for an autologous induced pluripotent stem cells‐based therapy.

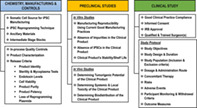

**Significance statement**

The induced pluripotent stem cells (iPSC) field has developed remarkably in the last decade, with some cell‐based therapies already in the clinic. However, there are still many hurdles to overcome before iPSCs attain their full clinical potential. Despite manufacturing challenges, autologous iPSC‐based cell therapies are being tested for various diseases. Clinical data from autologous stem cell therapies have suggested limited immune rejection and reduced necessity for postoperative immunosuppression. Autologous cell‐based therapies have their own set of regulatory requirements that need to be acknowledged and addressed to translate these products successfully to the clinic. A better understanding of an autologous stem cell therapy product and development of a robust manufacturing pipeline with safe and efficacious preclinical endpoints will help us develop reliable approaches to get autologous cell therapies commercially approved for unmet clinical needs in the near future.

## 1 INTRODUCTION

Cell‐based therapies are quickly becoming mainstream treatment modalities, thanks to recent successes with cancer immunotherapies in the U.S. and mesenchymal stem cell‐based therapies in Europe.^1^, ^2^, ^3^, ^4^, ^5^, ^6^, ^7^ Advances in protocols to efficiently differentiate pluripotent stem cells into various cell types have opened the possibility of developing a new class of cell‐based therapies—the replacement cell therapy.^8^, ^9^ Replacement cell therapy, as the name suggests, aims to replace degenerated or diseased tissue with a new “healthier” tissue derived from pluripotent stem cells. Two types of pluripotent stem cells are in use for developing replacement cell therapies—embryonic stem cells (ESCs) and induced pluripotent stem cells (iPSCs).^8^, ^10^, ^11^, ^12^, ^13^ Even though iPSCs were discovered almost a decade after ESCs, currently, iPSC use is gaining traction for developing cell‐based therapies. This is likely because, unlike ESCs that can only be used in allogeneic cell therapies, iPSCs provide the possibility of developing both allogeneic and autologous cell therapies, thus, providing an option of a personalized replacement therapy.^9^, ^14^, ^15^, ^16^, ^17^

Autologous cell therapy requires a new round of product manufacturing for each patient, which increases logistical challenges and costs associated with the manufacturing process.^18^, ^19^ But an advantage of autologous cell therapy is that the product engraftment in patients may not require the use of long‐term systemic immunosuppression as compared to an allogeneic cell therapy product that relies on the immunosuppression of patients to achieve longer‐term engraftment.^20^, ^21^, ^22^ Long‐term systemic immunosuppression is associated with serious adverse events, like an increased risk of infections or cardiovascular disorders—especially in older patients.^23^ Furthermore, immunosuppression discontinuation that may inadvertently happen in some patients will likely compromise graft survival.^22^, ^24^ This one key difference may significantly improve clinical outcomes of autologous cell therapy products as compared to allogeneic products and have favored the continued use of autologous products, despite their seemingly high cost. The approach for developing autologous and allogeneic iPSC‐based therapies is fundamentally different in several aspects, including the proof of concept, manufacturing workflow, preclinical study planning, regulatory approach, and the clinical strategy.^23^, ^24^ All of these differences affect the overall design of investigational new drug (IND)‐enabling studies, clinical trial design, market approval, financial feasibility, and commercialization strategies for autologous cell therapy products.

In the United States, the Center for Biologics Evaluation and Research at the FDA is responsible for regulating cell‐based therapies.^25^ The FDA has issued guidelines in the Code of Federal Regulations (CFR) for the development of products that it regulates. Multiple parts of Title 21 of the CFR provide general guidelines for the development of an iPSC‐derived product. The most critical of these include:

21 CFR Part 58—Good Laboratory Practice for Nonclinical Laboratory Studies

21 CFR Part 210 and 211—Current Good Manufacturing Practices

21 CFR 1271—Human Cells, Tissues, and Cellular and Tissue‐Based Products

21 CFR Part 312—Investigational New Drug Application.

These regulations explain guidelines for key aspects of cell‐based therapy development, for instance, donor screening, raw materials sourcing, documentation, vendor qualification, process and assay validation, preclinical studies, and product characterization. In summary, the standard pathway for any cell‐based product from bench to clinic is: (a) develop and characterize a prototype of the product such that it can be translated to a current Good Manufacturing Practice (cGMP)‐compliant manufacturing process; (b) conduct preclinical studies to demonstrate the safety and efficacy of the product; (c) compile an IND application that includes the manufacturing process in details, preclinical data and human study protocol, and submit it to the regulatory agency; (d) if the regulatory agency does not have any concerns to put the IND application on hold, start the phase I clinical trial to test product's safety in the target patient population; (e) once the initial safety profile of the cell therapy product is established in a phase I study, file IND application for the follow‐up trials to test product safety and efficacy in a larger population; (f) compile data from clinical trials to demonstrate product's safety and efficacy statistically and submit a Biologics License Application (BLA) to the regulatory agency to request for commercial approval of the cell‐based therapy product. For a checklist of the information required by the U.S. FDA to compile a phase I IND application, see Table 1, including required sections in (a) chemistry, manufacturing and controls, (b) non‐clinical information, and (c) clinical study plan. For details of an approval path for cell therapy BLA, see Creasey et al.^26^

**TABLE 1:** Typical requirements for an IND‐application for the U.S. FDA

CMC	Non‐clinical information	Clinical synopsis
Manufacturing site Equipment QC Sterility SOPs Product description Drug product Drug substance Flowchart for manufacturing workflow Detailed description of manufacturing process List of reagents Testing and specification Final formulation Manufacturing process development Product differentiation background Optimization description Protocol eepeatability Comparison of non‐Clinical and GMP runs Storage Shipping conditions	POC/efficacy studies Feasibility studies in pre‐clinical models POC and efficacy in pre‐clinical models Protocols for feasibility, POC/ efficacy Studies Proposed preclinical GLP toxicology and tumorigenicity studies Single‐dose toxicity Repeat‐dose toxicity Genotoxicity Carcinogenicity Reproductive and developmental toxicity Local tolerance (irritation, toxicity) Toxicokinetic Pharmacokinetics Method of administration Absorption Distribution Metabolism Excretion Pharmacokinetic drug interactions Analytical methods and validation reports Absorption after a single dose Organ distribution	Clinical phase Treatment indication Study objectives Study design Study population Test product Dosage Route of administration Treatment duration Criteria for evaluation Primary endpoints Secondary endpoints Study duration Study entry criteria Inclusion criteria Exclusion criteria Study assessments Statistical methods Assessments monitoring

Abbreviations: CMC, chemistry manufacturing and controls; GLP, good laboratory practice; GMP, good manufacturing practice; POC, proof of concept; QC, Quality control; SOP, standard operating procedure.

To date, none of the ESC‐ or iPSC‐based therapies have reached the stage of BLA submission for market approval. Presently, there are four FDA‐approved clinical trials in phase I/II in the U.S. testing iPSC‐derived products.^27^ The therapeutic effect of these iPSC‐based therapies is being evaluated for different diseases, including age‐related macular degeneration, advanced solid tumors including lymphoma, relapsed/refractory acute myeloid leukemia and B‐cell lymphoma, and chronic heart failure. Internationally, there are six additional iPSC‐derived products in clinical studies—in Japan, China, and Australia, for details see Martín‐Ibáñez and Sareen.^27^

With the continued development of autologous iPSC‐based therapies, there is a need to develop a regulatory roadmap for manufacturing and preclinical studies required to complete a phase I IND‐application. The challenges in the development process of autologous iPSC‐based products include establishing tissue and donor source, the heterogeneous phenotype of cells, elaborate manufacturing process, intricate in‐process quality controls, cryopreservation of intermediate and/or the final product, need for detailed product characterization, and short shelf life of a live product.^20^, ^23^ These challenges can markedly influence the cell therapy product profile and need to be adequately addressed in the early stages of product development. Additional manufacturing process controls involve validation of analytical testing methods employed for the in‐process and final product release assays, including assay specificity, sensitivity, accuracy, and reproducibility; this is essential to ensure batch‐to‐batch consistency and product comparability between multiple runs.^17^, ^24^ Establishing robust and standard practices around manufacturing, handling, delivery, shipping, and storage of the cell therapy product will help ease the path to market approval and also reduce manufacturing expenses in the long run. The main factor for the potentially higher cost of autologous cell therapy products as compared to allogeneic cell therapy products is the need to repeat the manufacturing process from start‐to‐finish for every patient. Manufacturing process repetition increases labor, facility operation, and consumable cost.^28^ Several of these costs can be lessened and controlled by the use of automation. Automated bioreactors and cell culturing robots combined with artificial intelligence‐based product analysis tools are being adapted for iPSC‐based therapies.^29^, ^30^, ^31^ Because of the smaller scale of manufacturing, autologous cell therapies are particularly amenable to scaled out automation. It is worth noting that since currently there is no commercially approved autologous or allogeneic iPSC‐derived cell therapy product, manufacturing cost can only be conceptualized.

Using our experience in developing a phase I/IIa IND application for the use of an autologous iPSC‐derived retinal pigment epithelium replacement tissue for macular degeneration patients, we discuss the most recent regulatory considerations for developing autologous cell therapy.^32^

## 2 CHEMISTRY, MANUFACTURING, AND CONTROLS

The chemistry, manufacturing, and controls (CMC) section is one of the most critical components of phase I autologous cell therapy IND application. It includes information about the product manufacturing process, characterization, in‐process and lot release testing, and stability. The product released from the GMP‐compliant manufacturing suite for patient administration is referred to as the Drug Product (DP). The DP consists of the cell product and its preservative, also known as an excipient. The primary objective of any regulatory agency is to assure the safety and rights of the subjects (details in 21 CFR 312.22(a) for the U.S. FDA). Thus, sufficient information should be provided in this section to ensure proper identification, characterization, control of quality, and purity of the DP. For a detailed organization of the CMC section in the IND application, refer to Table 2.

**TABLE 2:** Chemistry manufacturing and controls requirements for a phase I IND application

Drug product	Drug substance	Device
General information Description and composition Pharmaceutical development Components Formulation development Manufacturing Manufacturer Batch formula Control of critical process intermediates Control of excipients Control of DP Analytical procedure Validation of analytical procedures Stability of DP Manufacturing process and process controls Batch and scale Manufacturing process and process controls Reprograming and differentiation protocols Specifications for process intermediates Specification of cells Specifications of release assays	General information Manufacture Manufacturer Reprograming protocol Differentiation protocol Description of manufacturing process and process control Biological sourced reagents Control of materials Controls of critical steps Controls of intermediates Manufacturing process development Intermediate stage characterization Elucidation of structure Impurities Presence of iPSC in DP Residual reprograming vectors Residual medium components	Description of devices Description of all device Device schematic and components Manufacturing process Quality control Packaging and sterilization Device biocompatibility Transplant delivery device Surgical procedure and delivery device usage General procedure Performance testing and training Comparison to non‐clinical use Stability Biocompatibility tests Material safety data sheets for device parts

Abbreviations: DP, drug product.

### 2.1 Starting material

One of the first quality checks to be put in place for an autologous iPSC‐manufacturing pipeline is the starting material. In the case of an iPSC‐derived product, it is donor cells derived from blood, skin fibroblasts, or any other somatic cell type. Centers for Disease Control and Prevention (CDC) and the American Association of Blood Banks (AABB) recommend a list of specific disease pathogens for which any donor, including a patient, should be screened for before blood collection.^33^, ^34^ This guideline can help with autologous iPSC‐product manufacturing as well. Donors that test positive for any of the pathogens listed below may be excluded from the study. This exclusion ensures that these pathogens do not propagate from donor material into the manufacturing workflow and to other cell therapy products. The pathogens and tests for their detection listed by CDC and AABB are:

1. Rapid Plasma Reagin test for Syphilis
2. Flow immunoassay to detect anti‐Syphilis antibodies
3. QuantiFERON Gold immunoassay for *Mycobacterium tuberculosis*
4. Immunoassay to detect anti‐Hepatitis B surface antibodies
5. Immunoassay to detect anti‐Hepatitis B core antibodies
6. Immunoassay to detect anti‐Hepatitis C antibodies
7. Immunoassay to detect anti‐HIV 1/2 antibodies
8. Immunoassay to detect anti‐HTLV‐1/2 antibodies
9. RT‐PCR to detect HIV‐1/HCV/HBV nucleic acids
10. Immunoassay to detect anti‐*Trypanosoma Cruzi* antibodies
11. RT‐PCR to detect West Nile Virus nucleic acids
12. Immunoassay to anti‐West Nile Virus antibodies

In addition to testing patients for these well‐established pathogen panels, donors may also be tested for ongoing and highly virulent infections like COVID‐19. Most commonly used RT‐PCR‐based tests can even be performed on blood samples collected at home. See more details at (https://www.fda.gov/medical-devices/coronavirus-disease-2019-covid-19-emergency-use-authorizations-medical-devices/vitro-diagnostics-euas).

### 2.2 Cell source

iPSCs can be generated by reprogramming of any somatic cell.^35^ But for generating a cell therapy product, the starting cell source may be relevant. The ongoing clinical trials have mainly used skin fibroblasts and peripheral blood CD34+ cells, for ease of cell isolation, iPSC manufacturing, and the quality of derived iPSCs.^32^, ^36^ As of now, there is no regulatory guidance available for the choice of a given somatic cell type. CD34+ cells have been demonstrated to have a higher reprogramming efficiency as compared to terminally differentiated blood cells, likely because these cells are already in a stem cell state, and their chromatin is better poised to reprogram into a fully pluripotent state.^37^ This cell type has resulted in the development of a highly reproducible autologous iPSC‐manufacturing process.^32^ Although there is a relatively lower yield of CD34+ cells from peripheral blood as compared to the cord blood, peripheral blood is easily obtainable from any patient and provides one of the least invasive cell sources for autologous iPSC generation.^32^, ^38^ Moreover, GMP‐compliant protocols have been developed to expand CD34+ cells to a sufficient number required for the iPSC reprogramming process.^32^, ^39^ In conclusion, the choice of starting cell source is flexible for an autologous cell therapy product with certain advantages provided by CD34+ cells.

### 2.3 iPSC reprogramming technique

An essential requirement for the iPSC reprogramming technique used in a clinical manufacturing process is the reproducible and efficient generation of fully‐pluripotent iPSCs with zero genomic “footprint” (no leftover traces of reprogramming factors in the host genome). First‐ever reprogramming into iPSCs was performed using four transcription factors, OCT3/4, SOX2, KLF4, and c‐MYC, traditionally called the Yamanaka factors.^11^, ^12^, ^13^ These transcription factors were delivered using a retroviral system, a method that leads to the integration of reprogramming factors into the transduced cell's genome.^40^ Such a reprogramming system, if used in generating a cell therapy product, will significantly increase scrutiny for regulatory approval. However, the reprogramming field has been evolving fast, and presently several zero genomic footprint reprogramming methods are available, including episomal plasmids, Sendai virus, adenovirus, minicircles, and miRNA, mRNA or protein‐based overexpression of reprogramming factors.^41^, ^42^, ^43^, ^44^, ^45^, ^46^, ^47^, ^48^, ^49^ There is limited data on the cost and validation of these zero‐footprint reprogramming techniques, especially when used for clinical‐grade manufacturing, but they all seem to work well to generate iPSCs.^40^ Independent of the reprogramming method used, a critical requirement for this step is to demonstrate the loss of these reprogramming substrates (zero footprints) because the continued presence of such factors may increase the tumorigenic potential of the final product.

### 2.4 Ancillary materials

Ancillary materials (AMs) are reagents or components of media used during the manufacturing of the cell therapy product but are not intended to be a part of the final product. These materials may be chemical or biological entities. There are two main regulatory concerns with AMs: (a) lacking purity and/or imprecise concentration of a chemical/biologics affects manufacturing reproducibility; (b) the presence of a xeno‐product can introduce agents that may cause an infection or inflammation when the product is transplanted in the patient. United States Pharmacopeia (USP)‐grade chemicals meet regulatory standards for clinical‐grade manufacturing and alleviate concerns about purity and quality.^50^, ^51^ This makes pharmacopeia‐grade chemicals as the first and the safest choice of AMs for any clinical‐grade manufacturing protocol. If a pharmacopeia‐grade AM is not available, the second choice is a GMP‐compliant reagent. GMP‐compliant reagents provide access to complete documentation to ensure product sterility and traceability of the reagent manufacturing process.^50^ Most of this information is available on Certificate of Analysis and Certificate of Origin of each GMP‐compliant AM. These two documents should be inspected to confirm the purity and sterility of each batch of the raw material. In cases where AM contains the animal‐derived component, adventitious agent testing for each batch of AM may need to be performed (usually done by the vendor). Furthermore, it needs to be confirmed that AMs with animal‐derived components are from the countries categorized by World Health Organization for controlled transmissible spongiform encephalopathy and bovine spongiform encephalopathy.^50^ If a USP or GMP‐compliant AM is not available—AM may still be used in the human phase I trial, but such AMs need to be switched to at least a GMP‐compliant version before phase II.^52^ AMs are one of the essential components of an iPSC manufacturing process. The choice of a correct category of reagent is critical for accelerated regulatory approval.

### 2.5 Cryopreservation and intermediate stocks

Autologous iPSC‐derived product manufacturing can often extend from weeks to months.^8^, ^32^ The long manufacturing process increases risks of contamination and conflicts with the surgery schedule. For example, if the product is delivered live for surgery, its shelf‐life is likely less than a few days. If a patient's surgery needs to be rescheduled, it may require a re‐run of the entire manufacturing process in case there are no intermediate stage cryopreserved stocks available. Furthermore, for an autologous manufacturing process, if multiple iPSC clones are simultaneously manufactured, cryopreservation allows the selection of clones that meet quality control (QC) criteria. Some potential stages for cryopreservation of intermediate products include (a) donor cells; (b) early passage iPSCs; (c) iPSCs at the passage before differentiation starts; and (d) progenitor and/or immature cells during differentiation.^8^ It is important to note that these intermediate cryopreservation stages should be planned before pivotal preclinical IND‐enabling studies are conducted. This is done to ensure that the DP derived after cryopreservation at these intermediate stages can be tested in vitro and in vivo for safety and efficacy. Although cryopreservation of intermediate products is not a regulatory requirement, it helps de‐risk the manufacturing process, especially for cases where the DP is delivered live.

### 2.6 Product characterization and in‐process controls

Critical Quality Attributes (CQAs) are measurable properties of a cell therapy product that help better characterize the product. CQAs are especially helpful for autologous products because they help understand variability and its source within the manufacturing process, determine the allowable limit of variability from batch‐to‐batch, and control this variability. These attributes are also referred to as in‐process QC checks (tests performed in the intermediates stages of product manufacturing) and release tests (tests performed on the DP prior to its release for patient administration). The frequency of in‐process QC checks can be determined depending on the duration of the manufacturing protocol. Longer manufacturing processes should have a higher frequency of in‐process QC checks so that a failed manufacturing run can be identified as early as possible to avoid loss of resources on a failed run. In the case of compromised sterility, if the manufacturing run is not terminated, contamination may spread to other runs. Listed below are a few CQAs that can be used across different cell therapy products.

1. **Sterility tests**: Sterility tests are used to confirm the absence of bacteria, fungi, and mycoplasma. Instructions for USP <71> compliant product sterility testing can be obtained from FDA guidance document 21 CFR 610.12. For bacteria and fungi tests, samples are tested for aerobic and anaerobic contaminants for 14 days.^53^ Mycoplasma testing is often performed by qPCR. The following are some of the time points for sterility testing in an autologous iPSC‐product manufacturing process.
  - After the introduction of reprogramming factors in starting cells
  - At iPSC intermediate cryo‐stage
  - At the progenitor cell cryo‐stage and other potential intermediate stages
  - The DP—on and before the transplantation date

Critical regulatory note: an analytical study for method suitability testing of specific cell culture media used at each of these stages should be performed to validate sterility tests for specific media used in manufacturing.

**2. Endotoxin test**: Endotoxins are lipopolysaccharide components in the cell walls of Gram‐negative bacteria and can cause severe inflammatory reactions if introduced with the transplant.^54^ Endotoxins are most frequently present in glass‐ and plastic‐ware but can be easily tested using turbidimetric limulus amebocyte lysate assay up to the sensitivity of 0.01 endotoxin units (EU)/mL.^55^ The acceptable endotoxin level for a cell therapy product is variable depending on the route of administration, and strict guidelines must be followed to ensure the manufacturing process does not introduce endotoxins higher than the allowed limit. Refer to:
  - Guidance for Industry: Pyrogen And Endotoxin Testing: Questions And Answers
  - Guidance for Industry: Endotoxin Testing for current FDA recommended endotoxin limits for clinical products.^56^, ^57^
**Product identity**: Matching the identity of the product to their donor is a critical requirement for an autologous manufacturing process. The concern is an inadvertent mix up between different donor samples when the facility manufactures multiple patient samples simultaneously. Numerous methods are available for identity matching: human leukocyte antigens (HLA) typing, short tandem repeat polymorphisms test, and single nucleotide polymorphism test.^58^, ^59^, ^60^ Identity testing can be performed at various stages of the manufacturing process and compared to primary donor material to ensure no inadvertent mix‐up occurred. Some steps for this testing include the intermediate cryo‐stages (iPSCs and progenitor cells) and the DP. An identity test is a regulatory requirement for autologous products.
**iPSCs working stock qualification**: It is likely that for an autologous product, multiple iPSC clones are manufactured simultaneously to allow the possibility of selecting the “best” clone(s) for manufacturing ‐ clones that have been fully reprogrammed, have lost reprogramming factors, and have a stable genome. Carrying a set of iPSC clones to an intermediate stage will allow their qualification and selection for differentiation. Listed below are a few key properties to pick the most suitable clone(s) for differentiation.
  4.1 ***iPSC purity***: The purity of iPSCs provides information about how well a given clone may have been reprogrammed and is a critical feature that may determine iPSCs' ability to generate a high‐quality end product. Purity can be determined using flow cytometry for well‐known pluripotency markers like OCT4, SSEA4, and TRA1‐81 or using gene expression assays (qPCR, RNAseq, etc.).^14^ Although this is not a regulatory requirement, it determines end‐product quality. Therefore, depending upon the robustness of the differentiation process, one may want to set limits of iPSC purity that work for a given differentiation protocol in the manufacturing process.
  4.2 ***Loss of reprogramming systems***: Most of the currently available reprogramming systems qualify as zero‐genomic footprint^40^; however, considering the risk associated, the absence of the reprogramming plasmids should be confirmed in the product. Often this is also done at the iPSC intermediate working stock stage. This can be done by testing for the absence of plasmid(s), the virus(es), or any other construct(s) used for reprogramming. This assay can be performed using any commercially available qPCR‐based assays. The most critical aspect of this assay is to determine its lower limit of detection and ensure that the assay is sensitive enough to detect less than one copy of the reprogramming system per cell.
  4.3 ***Genomic stability***: Previous work has suggested that during the reprogramming and/or cell passaging process, iPSCs may become genomically unstable, acquire karyotypic abnormalities, and/or may copy‐number variations and mutations.^36^, ^61^, ^62^ All of these changes may cause the final product to become tumorigenic or acquire an unstable or incomplete phenotype. iPSC karyotyping can be checked using the G‐band karyotyping assay, and oncogenic mutation discovery is possible using targeted sequencing of cancer‐related genes commonly mutated or rearranged in human cancers.^63^ G‐banding results are interpreted based on significant historical data, but analysis of oncoexome data is tricky.^64^, ^65^ It is possible to analyze the oncoexome data by direct comparison to donor cells. Note that currently, there is no regulatory guidance available in the U.S. on what specific assays to perform to determine product genomic stability, the depth, and kind of analysis may vary from product to product. Despite lacking evidence to support the predictive potential of genomic stability assays to determine the safety of the end product, they do provide additional confidence in the quality and safety of the product.
**Progenitor cell qualification**: Assessing the quality of differentiation midway through the manufacturing process provides confidence in product consistency, identity, sterility, and/or potency. Again, this is not a regulatory requirement, but it may help save resources by helping avoid failed runs and reduce batch‐to‐batch inconsistencies. For qualification analysis, assays like the expression of progenitor markers, structural, and/or functional characteristics of the product may be used.
**Drug product qualification**: CQAs of the DP is likely the most important aspect of any cell therapy product. The more one can learn about the CQAs of the product before transplantation in patients, the easier it becomes to predict safety and to determine the potency of the product. Most functional CQAs for the final cell‐therapy product are usually product‐specific, so here we emphasize the standard CQAs of the product.
  6.1 ***Sterility, Endotoxin, and Product Identity Tests***: These attributes were discussed in detail in points 1, 2, and 3 above.
  6.2 ***Purity***: Cellular composition of the DP, including non‐desired cell types, especially pluripotent cells, is critical to be determined to confirm the purity of the product. The iPSC presence can be easily detected using flow or qPCR‐based assays.^66^ However, the presence of non‐desired non‐iPSCs is hard to determine as their lineage is also unknown. Assays like scRNAseq may be used to address this specific problem.^67^ It is well known that pure iPSCs are prone to teratoma formation, and a low number of iPSCs can form a teratoma.^68^, ^69^, ^70^ Because of this, it is important to determine the lower limit of detection of iPSC detecting assays.
  6.3 ***Viability***: It is critical to determine and report the percentage of viable cells administered to the patients. Transplant viability may affect potency and inflammation upon delivery. Viability can be determined using techniques like automated live cell count machines, flow cytometry, and imaging. FDA recommends a minimum of 70% cell viability.^71^
  6.4 ***Potency***: Potency of a cell therapy product is its capacity to alter the disease course. The potency of a product is related to its measured efficacy in vitro and in vivo in animal models. Efficacy assays will vary from product to product, and various techniques, including artificial intelligence, may be used to determine these potency assays.^31^, ^32^ Independent of the kind of assay, it is critical that the potency assay used is validated before the product reaches the phase II clinical trial.

## 3 PRECLINICAL STUDIES: PRODUCT SAFETY AND EFFICACY

For any IND‐application to be activated to test a product in patients, there is a regulatory requirement to confirm the safety, and if possible, its efficacy. These data are collected in preclinical studies, which preferably should be Good Laboratory Practice (GLP)‐compliant (details available in 21 CFR Part 58 of the FDA and in Table 3). If these studies cannot be GLP‐compliant, a justification for non‐compliance may be required. Preclinical studies include in vitro and in vivo data.^72^ in vitro studies are performed to qualify manufacturing process reproducibility, product purity and safety, functional characteristics, and stability. In vivo studies are performed to investigate product toxicity (local and systemic), tumorigenicity, and biodistribution.^72^ Lastly, In vivo, efficacy can be performed in a disease‐relevant animal model or a model that mimics disease conditions.^32^ Here, we provide an overview of some of the standard preclinical studies. Due to the product‐specific nature of efficacy studies, those will not be discussed in much detail here.

**TABLE 3:** Pre‐clinical study requirements for a phase I IND application

General study information	Test system and study design	Results
Study objective Study timetable Study initiation date Experiment start date Inlife start date Interim sacrifice Terminal sacrifice Experiment completion date Study completion date Regulatory Test Guidelines Protocol adherence Animal welfare, care, and use statement Major computer systems Application name Application function Monitoring Monitors and documents facility storage conditions Electronic notes (eNotes) Electronic communication systems Statistical analysis software Archive statement	Test system and study design Species and dose administration rationale Animal specifications and acclimation Environmental conditions, diet, and water Animal identification, Study assignment and retention Dose formulation Dose formulation procedures and dose analysis Sample collection and handling Implant analysis Cell viability Sample collection and handling Sample analysis and disposition Inlife procedures Dose administration Medication regimen Clinical observations Health monitoring Clinical examinations Body weights Clinical laboratory procedures Clinical pathology Sample collection and handling Hematology, clinical chemistry, and urinalysis Bone marrow smear Terminal procedures Animal fate—dosed extras and animals not dosed Necropsy, organ weights, macroscopic observations, microscopic observations Biodistribution Data evaluation and statistical analysis	Dose analysis Cell identity Cell viability Cell potency Morphological evaluation Functional analysis Clinical observations Body weights Food consumption Veterinary treatments Target organ examinations Clinical laboratory evaluations Clinical pathology Scheduled and unscheduled euthanasia clinical pathology Terminal evaluations Mortality Organ weights Macroscopic observations Microscopic observations and immunohistochemistry Biodistribution Conclusion

### 3.1 Reproducibility of the manufacturing process and product characteristics

For autologous cell therapy, the process is the product, that is, confirming the manufacturing process reproducibility is a crucial part of the preclinical studies.^73^, ^74^ Once the research‐grade process is translated into a GMP‐facility, successful cGMP‐compliant manufacturing of the proposed clinical product needs to be demonstrated in the IND application, preferably from multiple patients. This exercise serves multiple purposes: it helps set release criteria that are widely applicable for the product derived from multiple patients; it helps train operators on the cGMP‐compliant manufacturing process; it helps better understand the range in which product CQAs fall when the product is manufactured from different patients; it helps define SOPs for a cGMP‐complaint process; and it helps manufacture sufficient product for preclinical studies.

### 3.2 Removal of impurities

Often, cell therapy products are cultured in media that contain recombinant proteins, chemicals, buffers, and serum (or cryo‐protectant if delivered frozen to the surgery suite). Such impurities can cause inflammation or toxicity systemically or at the site of transplantation. Therefore, the removal of such impurities may be required before transplantation.^71^ This can be easily achieved by several sequential washing steps. Removal can be demonstrated by calculation of the amount of a given impurity after subsequent dilutions and/or by specific assay like mass spectrometry.

### 3.3 In vitro safety

Leftover iPSCs in the DP is a major concern of regulatory authorities. Besides demonstrating the absence of iPSCs in the DP, in‐vitro “spiking” studies can be performed to demonstrate the non‐survival of iPSCs in the differentiation process. This assay is based on the hypothesis that iPSCs require special culture medium and cannot grow in a culture medium that includes targeting product‐specific differentiation factors. Following test groups can be used in the assay (a) 100% target cells, (b) 100% iPSCs (positive control), (c) 99% target cells mixed with 1% iPSCs, and (d) 90% target cells mixed with 10% iPSCs. Cells in these four groups are cultured using target product differentiating conditions.^32^ Techniques like flow cytometry, qPCR, and scRNAseq may be performed to determine surviving iPSC or sporadically formed cells of a different lineage. This assay provides additional confidence in the safety of the cell therapy product.

### 3.4 In‐vitro stability of the clinical product

After washing the cell culture medium, an excipient is added to the cell therapy product to act as a preservative prior to the release of the DP from the GMP‐compliant manufacturing suite. This excipient is to be used to store, transport, and administer the product. Thus, the choice of the excipient is very crucial to ensure that it is compatible with the cells and, importantly, is permitted or approved by the FDA to be administered in humans. One of the options to use as an excipient is isotonic saline. However, live cell therapy products may have a relatively short shelf life in the excipient used for dose administration and transport. Thus, it is crucial to determine the duration for which the clinical product is stable with optimal cell viability in the excipient, and in the delivery device. Product stability should be determined in its transportation container system and when loaded inside the transplantation device (Table 3).^71^ This study gives surgeons confidence about the product's shelf life while they prepare the patient for the surgery.

### 3.5 Preclinical toxicity and biodistribution

One of the main concerns for any new cell therapy product is its safety profile ‐ this includes non‐teratogenic/tumorigenic potential, any local or systemic toxicity, and non‐targeted migration of the transplanted cells.^21^, ^75^, ^76^, ^77^, ^78^ A phase I IND‐application of a stem cell‐derived product may not be approved without sufficient data on these three characteristics of the product. Different animal models can be used in preclinical studies to ensure that the transplanted human cells (xenograft) survive long enough to reveal their tumorigenic potential.^79^ It is the sponsor's responsibility to justify the suitability of chosen animal models based on the test article route, site of administration, its dosage, and long‐term survival. Preclinical studies need to be conducted using the dosage and delivery route that is representative of the regimen to be used in patients. The product in preclinical studies should be manufactured using the same manufacturing process, which will be used for product manufacturing during the clinical trial to demonstrate that the product proposed to be transplanted in humans has been thoroughly investigated in in‐vitro studies and animal models.

Furthermore, for an autologous iPSC‐derived product, cells derived from multiple (2 or more) donors may need to be tested in animals. One of the most critical requirements for such preclinical studies is that they should be GLP‐compliant with prospective study plans. Refer to Table 3 for an outline of the GLP‐compliant preclinical study design for an autologous iPSC‐derived cell‐therapy product.

It is worth noting that although preclinical animal testing can de‐risk an iPSC‐based cell therapy product to some extent, it cannot ascertain that the safety profile of human cells obtained from transplantation performed in animals will actually translate to patients. Therefore, prospective risk‐assessment and risk‐management of cell therapy products are quint‐essential. This may be done by a justification of dosage, delivery site, delivery route, disease stage, combined with data about the purity of the DP. If the DP is composed of post‐mitotic cells of only one lineage and is relatively free of non‐desired cells, safety risk associated with cells lessens significantly. But for products that contain a mixed population or stage of cells, the progenitor stage may contain pluripotent or multipotent cells. Teratoma formation has been detected with as low as 245 pluripotent cells.^80^ Thus, for products with a mixed population or stage of cells, a prospective risk‐management may be required in the clinical protocol, despite a demonstrated safety profile in preclinical studies.

### 3.6 Clinical considerations

A detailed discussion on clinical considerations for cell‐based products is beyond the scope of this article because of the uniqueness of clinical aspects of different disease indications. Patient safety is of paramount importance, and, in part, it is ensured by an institutional review board (IRB) and data safety monitoring board, in addition to the FDA approval of the IND‐application. To maintain the legitimacy of the trial, patients must not be incentivized or coaxed into the trial; rather, they should be enrolled using an IRB‐approved informed consent form. Because a phase I study by design is a safety trial, the first patient cohort should be chosen such that if the drug product fails its safety profile, it causes minimal or no harm to patients. Patients must be clearly informed of the potential risk associated with the first‐in‐human procedure.^81^

#### *CONCLUSION*

The iPSC field has developed remarkably in the last decade, with some cell‐based therapies already in the clinic. However, there are still many hurdles to overcome before iPSCs attain their full clinical potential. Despite manufacturing challenges, autologous iPSC‐based cell therapies are being tested for various diseases. Clinical data from autologous stem cell therapies have suggested limited immune rejection and reduced necessity for postoperative immunosuppression. Autologous cell‐based therapies have their own set of regulatory requirements that need to be acknowledged and addressed to translate these products successfully to the clinic. A better understanding of an autologous stem cell therapy product and development of a robust manufacturing pipeline with safe and efficacious preclinical endpoints will help us develop reliable approaches to get autologous cell therapies commercially approved for unmet clinical needs in the near future.

## CONFLICT OF INTEREST

The authors declared no potential conflict of interest.

## AUTHOR CONTRIBUTIONS

J.S.B, F.M., and B.K. contributed to the writing of the manuscript. B.K. supervised the content of this work.

## Data Availability

Data sharing is not applicable to this article as no new data were created or analyzed in this study.
